# Turgor loss point and vulnerability to xylem embolism predict species‐specific risk of drought‐induced decline of urban trees

**DOI:** 10.1111/plb.13355

**Published:** 2021-10-27

**Authors:** F. Petruzzellis, E. Tordoni, A. Di Bonaventura, M. Tomasella, S. Natale, F. Panepinto, G. Bacaro, A. Nardini

**Affiliations:** ^1^ Dipartimento di Scienze della Vita Università di Trieste Trieste Italia; ^2^ Dipartimento di Scienze agroalimentari, ambientali e animali Università di Udine Udine Italia; ^3^ Institute of Ecology and Earth Science University of Tartu Tartu Estonia; ^4^ Unità Tecnica Alberature e Parchi Servizio Strade e Verde Pubblico Comune di Trieste Trieste Italia

**Keywords:** Climate change, embolism, drought, tree mortality, turgor loss point, urban trees

## Abstract

- Increasing frequency and severity of drought events is posing risks to trees' health, including those planted in urban settlements. Drought‐induced decline of urban trees negatively affects ecosystem services of urban green spaces and implies cost for maintenance and removal of plants. We aimed at identifying physiological traits that can explain and predict the species‐specific vulnerability to climate change in urban habitats.
- We assessed the relationships between long‐term risk of decline of different tree species in a medium‐sized town and their key indicators of drought stress tolerance, *i.e*. turgor loss point (TLP) and vulnerability to xylem embolism (*P*_50_).
- Starting from 2012, the study area experienced several summer seasons with positive anomalies of temperature and negative anomalies of precipitation. This trend was coupled with increasing percentages of urban trees showing signs of crown die‐back and mortality. The species‐specific risk of decline was higher for species with less negative TLP and *P*_50_ values.
- The relationship between species‐specific risk of climate change‐induced decline of urban trees and key physiological indicators of drought tolerance confirms findings obtained in natural forests and highlights that TLP and *P*_50_ are useful indicators for species selection for tree plantation in towns, to mitigate negative impacts of climate change.

## Graphical abstract

The risk of decline and mortality of urban trees facing increased frequency/intensity of drought events was found to be correlated to species‐specific physiological traits, an in particular to their vulnerability to xylem embolism.

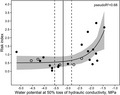

## INTRODUCTION

Climate change is posing new and extraordinary challenges to ecosystems and societies, calling for strategies of adaptation to global change impacts on natural habitats, as well as on human activities and well‐being (Fedele *et al*. 2019). Urban areas are recognized as major hotspots of global warming (Chapman *et al*. 2017) because of the intrinsic nature of urban development based on the pervasive substitution of natural vegetation and habitats with impermeable concrete surfaces. Urban areas are prone to more severe impacts of rising average temperatures and anomalous heatwaves due to the well‐known urban heat island (UHI) effect (Mohajerani *et al*. 2017). The thermal properties of urban surfaces, coupled with urban geometry and heat production associated with energy consumption, lead to air temperatures of 5 °C up to 15 °C higher than surrounding countryside, where natural vegetation assures substantial cooling *via* surface shading and evapotranspiration processes (Zeng *et al*. 2017). The UHI implies a strong reduction in thermal comfort for inhabitants of cities, which currently exceeds 50% of the global population (Grimm *et al*. 2008), translating into impacts on human health (Mika *et al*. 2018) and associated social and economic costs.

Among the possible adaptation strategies to excessive climate warming in cities, urban greening emerges as the most natural and logical one (Bowler *et al*. 2010) and has been proven to provide the desired benefits in buffering temperature peaks and mitigating social and sanitary impacts of UHI (Donovan *et al*. 2013; Chen *et al*. 2014; Edmondson *et al*. 2016; Zhang 2020). Increasing the extension of urban green areas may be problematic because of the extension of paved impermeable surfaces coupled with conflicting needs in the use of available space. Novel approaches to mitigate this problem have widely considered the possibility of greening up cities by exploiting typically under‐utilized urban surfaces, like the roofs of buildings (Savi *et al*. 2013; Li *et al*. 2014). While green roofs can address some of the ecological and societal issues of urban areas, they most often fail to offer green spaces that can be ‘experienced’ by people, such as street trees and parks. Indeed, it is exactly such kind of urban green areas that provides the most adequate balance of climate mitigation, health improvement and societal benefits in terms of nature experience, aggregation, active life and so on (Donovan 2017; Turner‐Skoff & Cavender 2019). Urban forests and trees, however, are not exempt from the negative impacts of climate change (Gillner *et al*. 2014; Nitschke *et al*. 2017; Zhang & Brack 2021). Similar to natural forests, trees growing in cities have been experiencing increasing rates of crown die‐back and mortality as a consequence of higher frequency and intensity of anomalous drought and heatwaves (Helama *et al*. 2012; Savi *et al*. 2015). Drought‐induced decline of urban trees has obvious negative effects on their ecosystem services (Nowak & Greenfield 2018), while the related costs to be sustained for tree pruning and/or removal, or their socio‐sanitary impacts on populations are more difficult to quantify. Hence, selection of trees to be planted in urban settlements should take into account the species‐specific likelihood of survival under future climate scenarios. This, in turn, requires an adequate understanding of the drivers and proxies of species‐specific drought tolerance in urban habitats.

Tree survival under drought mainly depends on the maintenance of adequate cell hydration and turgor (Zhu *et al*. 2018), assured by root‐to‐leaf water transport (Nardini & Salleo 2000). When evaporative water loss exceeds the water supply capacity, the plant water content declines, leading to turgor loss and/or xylem embolism when species‐specific water potential thresholds are surpassed. Prolonged turgor loss can lead to membrane disruption (Savi *et al*. 2016; Mantova *et al*. 2021) and cell death, potentially leading to irreversible plant decline. On the other hand, the progressive drop in xylem pressure can trigger xylem embolism (Sperry & Tyree 1988), leading to plant hydraulic failure (Sevanto *et al*. 2014). Indeed, the distribution of woody species along water availability gradients at different spatial scales is well correlated to their turgor loss point (TLP; Bartlett *et al*. 2012a; Nardini & Luglio 2014; Savi *et al*. 2017a; Kunert *et al*. 2021) and vulnerability to xylem embolism, generally expressed as the xylem water potential that induces 50% loss of xylem hydraulic conductivity (*P*_50_) (Trueba *et al*. 2017; Oliveira *et al*. 2019). It is also known that during periods of anomalous drought, in terms of duration and/or intensity, species with lower TLP and *P*_50_ have higher chances of surviving (Nardini *et al*. 2013; Maréchaux *et al*. 2015; Powell *et al*. 2017). Hence, TLP and *P*_50_ emerge as functional traits with a strong linkage to species‐specific drought tolerance and might be used as reliable and objective criteria to select urban tree species better suited to ongoing climate changes, especially in the case of hard surfaces where pavement features exacerbate the impact of seasonal drought on trees (Sjöman & Nielsen 2010; Morgenroth *et al*. 2013; Savi *et al*. 2015; Fini *et al*. 2017; Wang *et al*. 2019).

Despite the importance of water relations functional traits for survival of trees under future climate scenarios (Watkins *et al*. 2021), examples of their predictive value in terms of tree performance in urban settlements are still scarce. Sjöman *et al*. (2015) analysed the seasonal variation of TLP in 27 *Acer* genotypes widely used as street trees, revealing a wide range of tolerance to water deficits, with important consequences for the potential of species to tolerate periods of low water availability. In a further study, Sjöman *et al*. (2018) showed that TLP values of 45 urban tree species were correlated with a ‘drought tolerance score’ based on expert assessment by professionals involved in urban green management. Esperon‐Rodriguez *et al*. (2020) have recently reported that urban trees successfully growing in the warmest and driest sites of the Greater Sidney region had lower TLP than those occurring in the cooler and wetter areas. Data on species‐specific vulnerability to xylem embolism of urban trees, and its eventual role in the enhancement of drought tolerance of plants in urban settlements, are even more scant. Savi *et al*. (2015) have shown that trees growing in sites with extensive impermeable pavements are more vulnerable to xylem embolism than conspecific individuals growing in more natural sites, leading to reduced safety margins against drought‐induced catastrophic hydraulic failure. Litvak *et al*. (2012) showed that urban trees with higher (less negative) *P*_50_ had a more rapid reduction of stomatal aperture in response to increases in vapor pressure deficit (VPD), indicating that species more vulnerable to embolism might be not only prone to hydraulic dysfunction, but also less valuable in terms of evaporative cooling capacity during the warmest days of the year.

Despite these research efforts, evidence for a role of low TLP and/or *P*_50_ in preventing long‐term decline of urban trees under changing climatic conditions is still lacking. In 2012, the Municipality of Trieste (northeast Italy) started a survey that aimed to monitor urban tree health status within the municipal area. Over the last 15 years, the area has repeatedly experienced anomalous summer droughts and heatwaves that have caused extensive dieback and mortality of trees in natural forests (Nardini *et al*. 2013, 2016; Petrucco *et al*. 2017; Savi *et al*. 2019) and also in urban trees. In this study, by measuring TLP and *P*_50_ of most urban tree species growing in this area, we aimed to test the eventual relationships between the retrospective long‐term risk of decline of urban trees, and their physiological traits related to drought tolerance.

## MATERIAL AND METHODS

### Study area

The study area is located in the municipality of Trieste (Italy), a middle‐sized town (ca. 200,000 inhabitants) on the Adriatic coast with an urbanized area of about 28 km^2^ (Martini, 2006). Green areas occupy ca. 4% (Savi *et al*. 2015) of the city area, and host different plant species representing a total of 122 vascular plant families (Martini 2006). The climate of Trieste is transitional between the Mediterranean and Central European types and is characterized by cold winters and relatively dry periods in December–February and July–August (Savi *et al*. 2015). Mean annual temperature averages 15.9 °C, while annual rainfall totals 870 mm (www.osmer.fvg.it, accessed April 2021). Daily mean air temperatures and cumulative precipitation of the warmest quarter (June, July and August) of each year from 1994 to 2019 were retrieved from a weather station within Trieste municipal area (Molo Bandiera, www.osmer.fvg.it) to calculate climate anomalies between 2012 and 2019. Specifically, temperature and precipitation anomalies were calculated as:

(1) ΔT=Ti‐Tref

where *T i* is mean *T* in the *i*‐th year and *T* *ref* is mean *T* in the reference period (1994–2019).

(2) △Precipitation=((Pi‐Pref)/Pref)×100

where *Pi* is the cumulative precipitation in the *i*‐th year and *P* *ref* is mean cumulative precipitation in the reference period (1994–2019).

### Database of urban tree species and health status

Plant species included in the study were selected based on the database provided by Unità Tecnica Alberature e Parchi of the Municipality of Trieste. The database contains the coordinates of each tree planted within the city area (25,176 trees in total) as well as information about the risk of falling for each tree monitored from 2012 to the present, assessed by Visual Tree Assessment (VTA; Mattheck & Breloer 1994; Fink 2009) by independent trained experts. VTA was addressed also at evaluating the ‘risk to fall’ of trees, based on anamnesis (identifying symptoms, damage, defects and other anomalies that have direct or indirect repercussions on the stability of the tree or part of it), analysis (characteristics of the rooting site and peculiarities of the station, historical data on previous situations) and stability assessment (visual analysis eventually integrated with instrumental insights on the basis of the symptoms found). Most of the VTA evaluations were done on a different set of individuals each year. We found only 81 VTA evaluations (out of more than 15,000 total evaluations) related to the same individuals. In these cases, we included VTA evaluations on the same individual in the analyses only when the ‘risk to fall’ class changed. Table 1 reports the risk classes resulting from the VTA analysis, whose values spanned from class A (negligible risk to fall) to class D (extreme risk). We selected 32 species representing approximately 84% of the total trees contained in the database (Table 2). For each selected species, we calculated the relative frequency of occurrence in VTA classes as:

(3) Ni/NVTA total

where *Ni* is the number of individuals in the *i*‐class and *N* VTA total is the total number of VTA evaluation for the selected species.

**Table 1:** The VTA classes and associated definitions according to Società Italiana di Arboricoltura (SIA, isaitalia.org).

class	risk	definition
A	Negligible	Trees in this class do not have significant visual symptoms at the time of investigation, thus indicating that the health status has not deteriorated. For these individuals, a periodic visual inspection at no more than 5 years is recommended.
B	Low	Trees in this class have mild visual or instrumental (according to technician’s opinion) symptoms at the time of investigation, indicating that tree safety had not significantly deteriorated. For these individuals, a periodic visual inspection, at intervals established by the technician in charge, but not longer than 3 years, is recommended. Any instrumental diagnostic investigation and its frequency are at the discretion of the technician.
C	Mild	Trees in this class have both visual and instrumental significant symptoms at the time of investigation, indicating that tree safety has significantly worsened. For these individuals, a periodic visual inspection, at intervals established by the technician in charge but not longer than 2 years, is highly recommended. Any instrumental diagnostic investigation and its frequency are at the discretion of the technician. For these individuals, the technician can plan management activities to reduce the danger level and change the VTA class.
C/D	Severe	Trees in this class have both visual and instrumental significant severe symptoms at the time of investigation, indicating that tree safety has dramatically worsened. For these individuals, management activities planned by the technician in charge are mandatory and must be implemented to reduce the danger level and be compatible with good arboricultural practices. According to the feasibility of these activities, the VTA class could be changed. When management activities cannot be applied, the tree should be assigned to VTA class D.
D	Extreme	Trees in this class have both visual and instrumental significant severe symptoms at the time of investigation, indicating such trees are no longer safe. For these individuals, any management activity compatible with good arboricultural practices would not improve tree safety. Hence, these individuals must be cut down.

Please note that in this study, C/D class was merged with D class.

VTA = Visual Tree Assessment.

**Table 2:** List of the species included in this study, along with the ratio between number of individuals occurring in A or B classes and in C or D classes (AB/CD), and total number of VTA of each sampled species.

	species	total number of trees	A	B	C	D	risk index	number of VTA
Angiosperms	*Styphnolobium japonicum*	124	0.04	0.37	0.53	0.05	2.88	248
	*Populus nigra*	105	0.03	0.39	0.56	0.02	2.61	488
	*Robinia pseudoacacia*	795	0.06	0.43	0.49	0.02	2.35	1307
	*Aesculus hippocastanum*	764	0.04	0.49	0.47	0.01	1.59	2531
	*Quercus pubescens*	169	0.03	0.53	0.41	0.03	1.58	359
	*Tilia cordata*	206	0.62	0.19	0.18	0.02	1.30	255
	*Juglans regia*	136	0.08	0.59	0.32	0.01	1.19	232
	*Prunus mahaleb*	122	0.10	0.66	0.24	0.00	1.15	127
	*Celtis australis*	1249	0.14	0.54	0.31	0.01	1.08	2941
	*Cercis siliquastrum*	153	0.16	0.55	0.29	0.00	1.05	170
	*Prunus cerasifera*	109	0.41	0.45	0.14	0.00	1.00	136
	*Quercus ilex*	738	0.25	0.51	0.24	0.01	0.83	1528
	*Tamarix gallica*	231	0.03	0.50	0.46	0.00	0.68	436
	*Ulmus minor*	289	0.20	0.60	0.21	0.00	0.68	289
	*Platanus* × *acerifolia*	2747	0.06	0.67	0.26	0.01	0.63	8362
	*Acer campestre*	631	0.45	0.44	0.11	0.00	0.61	822
	*Fraxinus ornus*	582	0.22	0.65	0.12	0.01	0.43	396
	*Ailanthus altissima*	72	0.00	0.67	0.33	0.00	0.42	51
	*Ostrya carpinifolia*	258	0.15	0.74	0.11	0.00	0.34	206
	*Tilia platyphyllos*	1435	0.30	0.57	0.13	0.00	0.34	2316
	*Laurus nobilis*	377	0.13	0.78	0.08	0.01	0.26	327
	*Ligustrum lucidum*	63	0.26	0.66	0.08	0.00	0.18	64
	*Carpinus betulus*	728	0.84	0.15	0.01	0.00	0.04	526
Gymnosperms	*Ginkgo biloba*	24	0.33	0.33	0.33	0.00	1.50	20
	*Pinus pinea*	839	0.01	0.71	0.28	0.00	0.77	1100
	*Cedrus deodara*	1735	0.04	0.64	0.32	0.00	0.72	3036
	*Cedrus atlantica*	455	0.02	0.71	0.26	0.00	0.63	996
	*Cupressus arizonica*	497	0.01	0.69	0.29	0.01	0.62	1081
	*Cupressus sempervirens*	1658	0.08	0.74	0.18	0.00	0.47	2440
	*Pinus nigra*	1857	0.07	0.80	0.12	0.00	0.39	2224
	*Pinus sylvestris*	310	0.00	0.83	0.17	0.00	0.39	357
	*Cedrus libani*	160	0.04	0.93	0.03	0.00	0.15	349

VTA = Visual Tree Assessment.

Moreover, we were interested in calculating an index summarizing the proportion of trees with at least a mild risk to fall for each species. Since the relative frequency of occurrence in a VTA class depends on the number of monitored trees (which was not equal for all the species), we calculated a Risk index representing the relative proportion of trees with at least a mild risk to fall as:

(4) Risk index=Number trees in C,C/D or D classes/number trees in A and B classes

The trees used for experimental measurements of functional traits were not irrigated, nor pruned or fertilized. Trees included in the VTA analysis were also not irrigated or fertilized, but some of them had been pruned over the last decade.

### Turgor loss point

In summer 2020, a sampling survey was conducted to collect leaves for measurements of leaf water potential at TLP. All the samples were collected in the timeframe between 11.00–15.00 h within 2 weeks, in order to exclude any possible confounding effect on the analyses derived from daily or seasonal osmotic adjustment. For each species, three healthy individuals were randomly selected in different locations with an optimal coverage and disposition across the study area with the aim of maximizing the variability accounted for spatial distance between individuals in different growing conditions. Three leaf pairs were randomly sampled from each individual and leaf dry matter content (LDMC) was measured on one leaf, while TLP was measured on another one. Twigs were detached, wrapped in cling film and put in plastic bags containing wet paper to avoid dehydration. Samples were stored in cool bags until processing in the laboratory within 2 h of sampling. Twigs bearing leaves were first rehydrated overnight and then LDMC and TLP measured following Petruzzellis *et al*. (2019). For LDMC, leaf turgid weight (without leaf petioles or rachis) was measured with an analytical balance immediately after the rehydration procedure. Leaves were then oven‐dried for 24 h at 70°C to obtain their dry weight. LDMC was calculated as:

(5) $$\text{LDMC}\;=\;\text{Leaf dry weight/Leaf turgid weight}\;\left({\text{mg g}}^{‐1}\right)$$

The TLP has been traditionally estimated from water potential isotherms, but the time‐consuming nature of this procedure has limited the inclusion of TLP in studies involving large numbers of species/individuals and/or study sites. In this paper, we used an alternative method to obtain TLP from measurement of the osmotic potential at full turgor (π_0_) by directly measuring the osmotic potential of sap extracted from leaf tissues using a thermocouple psychrometer (Bartlett *et al*. 2012b; Petruzzellis *et al*. 2019). Following this procedure, leaf samples were rapidly frozen in liquid nitrogen, which induces cell disruption and the release of symplastic content. After thawing, the osmotic potential at full turgor of leaf samples treated in liquid nitrogen could be measured with an osmometer or a dewpoint hygrometer (π_0_osm_). In this study, one leaf for each individual was roughly crushed and sealed in cling film immediately after rehydration. Note that for some species with small leaves and for gymnosperms, we had to use more than one leaf for each sample to obtain a significant amount of biomass. Samples were immersed in liquid nitrogen for 2 min, and leaves (still sealed in cling film) were carefully ground and stored in sealed plastic bottles at −20°C until measurements. Samples were thawed at room temperature for 5 min, while still sealed in cling film and in plastic bottles. Measurements of the osmotic potential at full turgor were done with a dew point hygrometer (π_0_osm_) (WP4; METER Group, Pullman, WA, USA). Because π_0_osm_ could be affected by dilution or enrichment of solutes of symplastic fluids (Bartlett *et al*. 2012b), the osmotic potential at full turgor (π_0_) was calculated from the following equations based on empirical analysis (Petruzzellis *et al*. 2019):

(6) $$\pi_{0}=0.506\pi_{0\_\text{osm}}‐0.00\text{2LDMC}$$

where π_0_osm_ is the osmotic potential at full turgor measured with the dewpoint hygrometer and LDMC is leaf dry matter content (expressed in mg g^−1^).

Finally, TLP was calculated as indicated in Petruzzellis *et al*. (2019):

(7) $$\text{TLP}\;=1.31\pi_{0}‐0.03$$

where π_0_ is the osmotic potential at full turgor estimated following Equation (6).

### Vulnerability to xylem embolism and wood density

Vulnerability to xylem embolism is generally assessed through the measurement of vulnerability curves (VC), which allow estimating the water potential inducing 50% loss of hydraulic conductivity (*P*_50_). In this study, we derived *P*_50_ values for our study species from the Xylem Functional Traits (XFT) database (Choat *et al*. 2012), integrated with more recent studies not included in XFT. In accordance with previous studies based on the XFT dataset (*e.g*. Trugman *et al*. 2020), we considered only *P*_50_ data measured in branches and we discarded measurements from r‐shaped vulnerability curves (Cochard *et al*. 2013). For species with more than one value, we considered the average value from different studies for subsequent analysis. A complete summary of *P*_50_ mean values of each species included in the study, along with the relative references, is reported in Tables S1 and S2.

Wood density (WD) was measured on the same individuals sampled for TLP measurements. For each individual, 2‐year‐old segments from three stems were sampled, immediately wrapped in cling film and stored in plastic bags placed in cool bags until measurement in the laboratory. WD was calculated as:

(8) $$\text{WD}\;=\;\text{Wood dry weight/Wood fresh volume}\;\left({\text{g cm}}^{‐3}\right)$$

Bark was removed from 3‐cm long segments and samples were rehydrated overnight in vials filled with tap water. Fresh volume was measured using a water displacement method (Petruzzellis *et al*. 2018) before oven‐drying the samples at 70°C for 24 h. Samples were then weighed to obtain wood dry weight.

### Statistical analyses

Simple linear regressions were fitted to test the relationship between the Risk index and the functional traits. Specifically, three independent models were fitted to estimate the Risk index as a function of TLP, LDMC or WD. Model’s assumptions were visually checked by means of residuals’ quantile‐quantile plots (for normality of residuals) and residuals *versus* fitted values plots (for homoscedasticity assumptions). In addition, the nonlinear relationship between the Risk index and *P*_50_ was assessed by fitting an exponential function. The Pseudo *R* ^2^ of the non‐linear model was estimated using the Nagelkerke method using *nagelkerke* function in the ‘rcompanion’ R package. The breakpoint of the relationship between the Risk index and *P*_50_ was assessed through *segmented* function in the ‘segmented’ R package.

## RESULTS

**Fig. 1:**
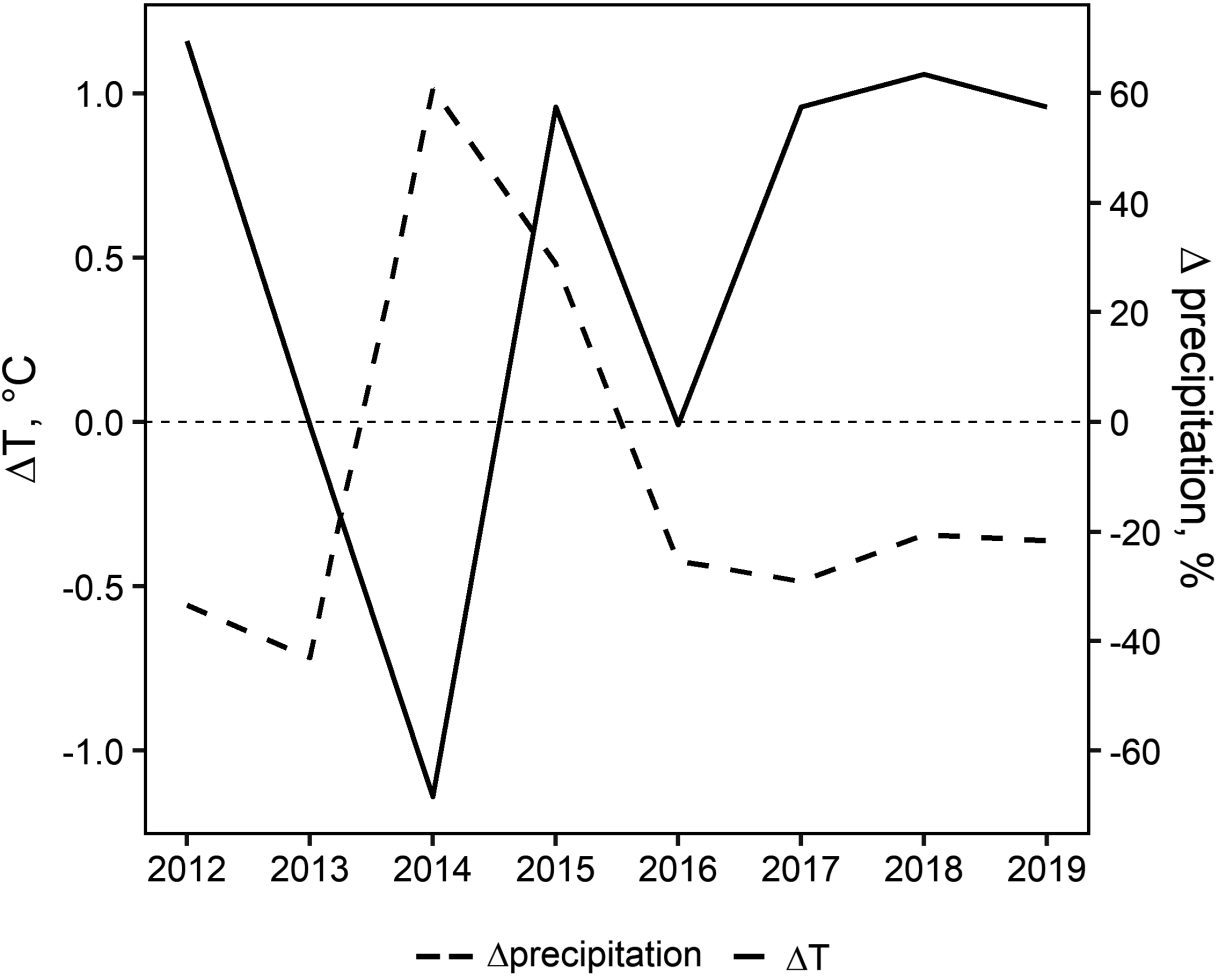
Trends in temperature and precipitation anomalies (Δ*T* and Δ precipitation, respectively) in the warmest quarter (June, July and August) of each year from 2012 to 2019.

Climate trends in the study area are shown in Fig. 1. Summer 2012 had the highest Δ*T* (+1.16°C) and one of the lowest ΔPrecipitation (−33.4%), while 2014 had the lowest Δ*T* (−1.14°C) and the highest ΔPrecipitation (+60.9%). In general, climate anomalies were more pronounced from 2015 to 2019, with Δ*T* > 0 and ΔPrecipitation < 0 in almost all years.

**Fig. 2:**
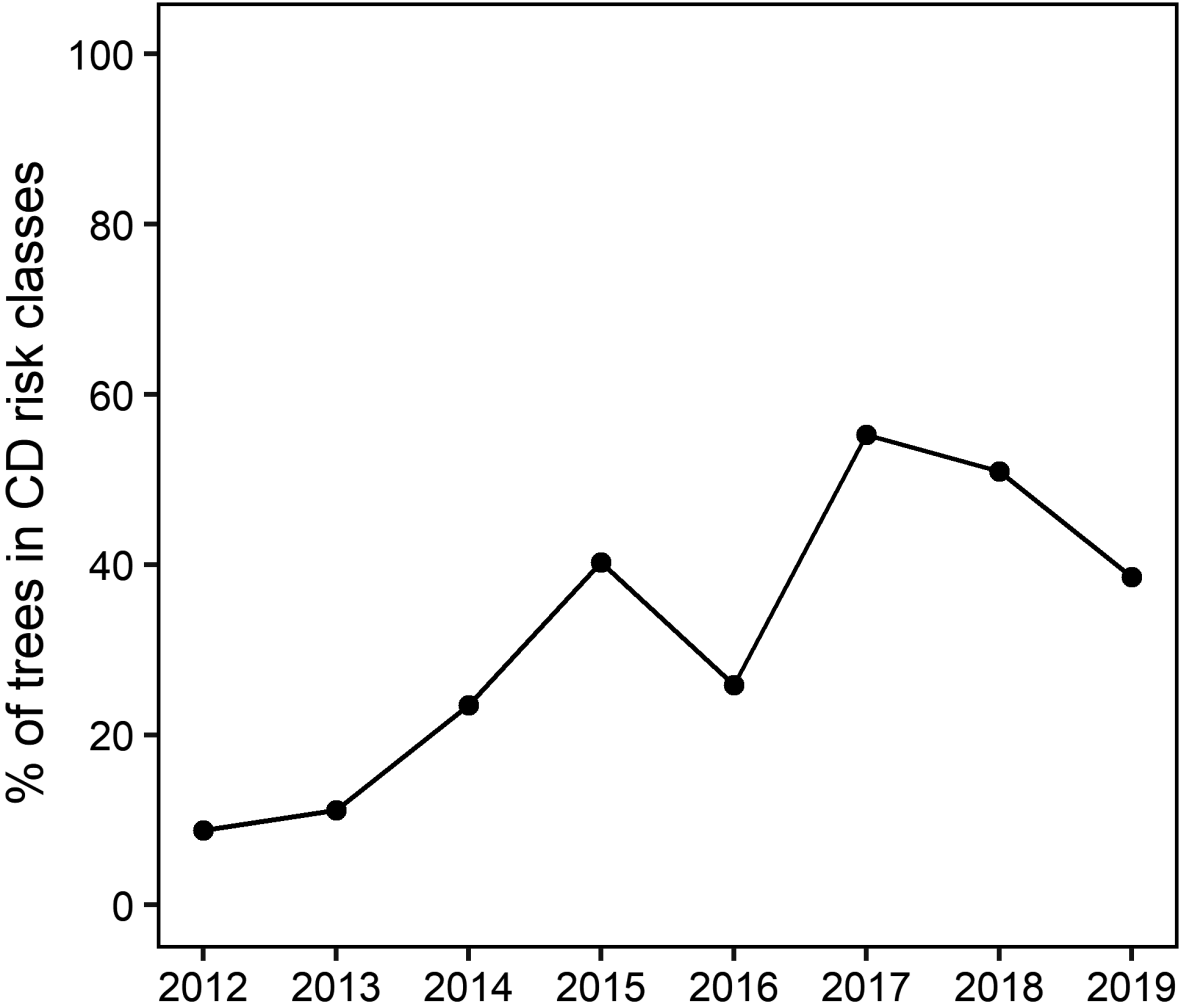
Trend in the percentage of trees reported in C, C/D or D Visual Tree Assessment (VTA) classes in each year from 2012 to 2019.

Table 2 summarizes the relative frequency of occurrence for each species in each VTA class along with Risk index values. In total, 13,447 VTA values were included in the database of urban tree species as well as health status from 2012 to 2020. Interestingly, the percentage of trees evaluated in vulnerable classes (*i.e*. C, C/D and D classes) has increased over the last 8 years, as depicted in Fig. 2. Overall, the number of VTA evaluations ranged between 20 (for *Gingko biloba*) and 8 (for *Platanus* × *acerifolia*) (Table 2). The minimum value of the Risk index was 0.04 (in *Carpinus betulus*), while the maximum was 2.88 (in *Styphnolobium japonica*).

Mean values and associated SD of LDMC, TLP, *P*_50_ and WD of each species are reported in Table S1. The LDMC ranged between 291 ± 39 mg g^−1^ in *Platanus* × *acerifolia* and 534 ± 40 mg g^−1^ in *Quercus ilex*. Minimum values of TLP were measured in *Q*. *ilex* (−3.09 ± 0.38 MPa), while the maximum values were measured in *Platanu*s × *acerifolia* (−1.58 ± 0.16 MPa). The lowest WD value was measured in *Tilia cordata* (0.32 ± 0.02 g cm^−3^), while *Cedrus deodara* had the highest value (0.83 ± 0.22 g cm^−3^).

**Fig. 3:**
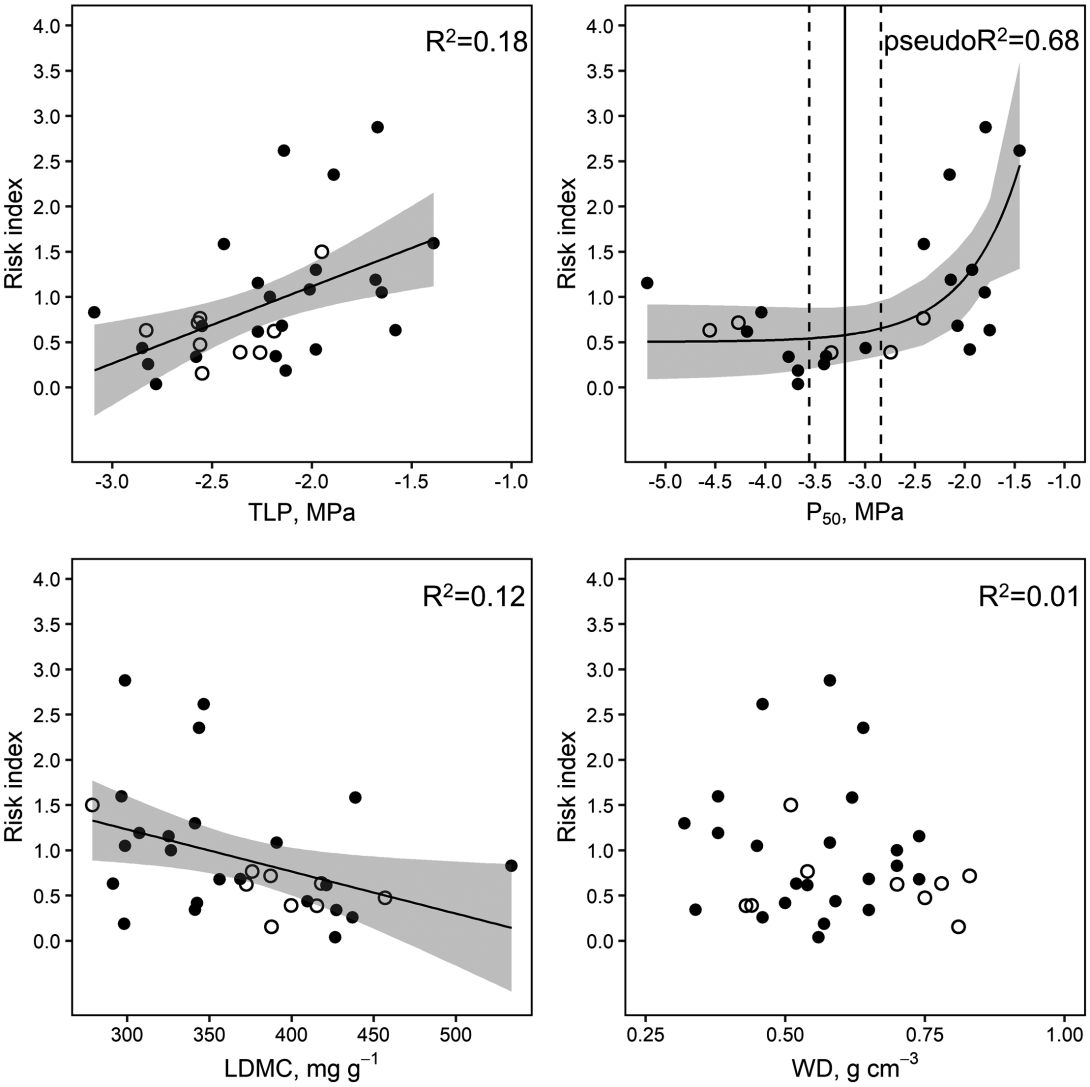
Relationship between Risk index and turgor loss point (TLP, upper left panel), *P*_50_ (upper right panel), leaf dry matter content (LDMC, lower left panel) and WD (lower right panel) as measured in the species selected in this study and retrieved from literature data, respectively. Solid and open circles represent angiosperm and gymnosperm species, respectively. Solid lines represent the overall regression trends and shaded areas represent 95% confidence intervals. The vertical solid line in the upper right panel represents the break point of the relationship between risk index and *P*_50_, while the dashed black lines represent its standard error (SE).

We were able to retrieve data of *P*_50_ for 22 species out of 32 (Tables S1 and S2). Specifically, the highest value was found in *Populus nigra* (−1.5 MPa), while *Prunus mahaleb* had the lowest value (−5.2 MPa). TLP and *P*_50_ values were found to be closely correlated. A significant positive relationship was found between the Risk index and both TLP and *P*_50_ (Fig. 3; Table S3), while a negative correlation was found between the Risk index and LDMC (Fig. 3; Table S3). No significant relationship was observed between the Risk index and WD (Fig. 3; Table S3). The breakpoint of the relationship between the Risk index and *P*_50_ was −3.2 ± 0.36 MPa (Table S4).

## DISCUSSION

Across 32 tree species growing in the town of Trieste, and representing more than 80% of the total number of urban trees, we found significant correlations between the species‐specific decline over the last 8 years, and two water relations traits correlated to drought tolerance, *i.e*. TLP and *P*_50_. This suggests that these parameters are reliable proxies for selection of trees that are more tolerant to the projected increase in drought frequency and severity, and thus should be preferred for urban greening interventions in drought‐prone areas. At the same time, the relationships found in this study offer a tool to anticipate the identification of trees that will potentially undergo crown dieback and decline over the next decades, calling for the need to carefully evaluate their health status to prevent accidental fallings of branches or whole trees.

Trees growing in urban areas are frequently exposed to levels of water stress well above those experienced by conspecific individuals in natural habitats (Gillner *et al*. 2017; Pretzsch *et al*. 2017; Meineke & Frank 2018). This is because urban trees frequently grow in sites covered by impermeable pavements, enhancing run off with consequent poor water storage in soils (Morgenroth *et al*. 2013). Moreover, impermeable surfaces also reduce gas exchange between the soil and atmosphere, limiting root growth and metabolism and eventually limiting root water uptake (Viswanathan *et al*. 2011; Volder *et al*. 2014) and photosynthesis rates (Wang *et al*. 2019). Edaphic drought in urban areas is somewhat site‐specific (Savi *et al*. 2015), but other environmental factors are more pervasive and act as enhancers of water stress experienced by urban trees. This is the case of VPD, which increases disproportionately at increasing temperatures driven by the UHI effect (Grossiord *et al*. 2020).

Consistent with observations of tree crown die‐back in the natural forests surrounding the town of Trieste, triggered by an extreme drought and summer heatwave in 2012 (Nardini *et al*. 2013; Petrucco *et al*. 2017), trees in the urban area showed signs of increasing decline, starting from 2013. In fact, the percentage of trees showing signs of significant damage or completely dead was relatively low in 2013, but started to increase thereafter, peaking at 60% in 2017. Just as in natural settings, not all urban tree species showed signs of impact of increasingly drier conditions, with some species maintaining an overall good health status and others showing severe damage at population level. As an example, *C*. *libani* and *Ligustrum lucidum* suffered very low impacts and were classified in a low‐risk class. On the other hand, species like *P*. *nigra* and *S*. *japonica* fell into a very high‐risk category of increased crown die‐back and decline over the long‐term survey.

Species‐specific risk of tree decline at population level turned out to be related to both TLP and *P*_50_, but not to WD. Correlations between TLP and plant performance under drought have been frequently reported for trees growing in natural habitats (Bartlett *et al*. 2012a; Binks *et al*. 2016; Kunert *et al*. 2021) and were recently suggested to hold true also for urban trees (Sjöman *et al*. 2015, 2018). However, our dataset is the first direct evidence that tree species with lower TLP are more tolerant to climate extremes in an urban settlement. Lower TLP allows plants to maintain turgor at lower water potential compared to species with relatively higher values of this functional trait. Under moderate drought stress conditions, maintenance of higher turgor values allows plants to keep stomata open to fuel photosynthetic processes, thus maintaining an optimal content of non‐structural carbohydrates to sustain water transport, growth, reproduction and defence against pathogens (Anderegg & Callaway 2012; Sapes *et al*. 2021; Tomasella *et al*. 2021). Even under severe drought leading to stomatal closure, low TLP would delay the risk of cytorrhyzis and membrane disruption, which ultimately lead to cell death (Guadagno *et al*. 2017; Mantova *et al*. 2021). The correlation between tree damage levels and TLP found in this study offers strong support to the hypothesis that low TLP has an adaptive value for urban trees under progressive global warming. At the same time, our findings suggest that this parameter could be used as a reliable proxy of species‐specific drought tolerance that could be adopted for screening and selection of tree species better suited to urban sites with pronounced edaphic or atmospheric aridity, and more likely to survive the increasing intensity and frequency of droughts and heatwaves in urban areas of the globe (Perkins‐Kirkpatrick & Lewis 2020). It should be noted, however, that some species in our dataset were characterized by relatively high TLP, and yet scored quite low among the risk index classes. This is the case of *Platanus* × *acerifolia*, and it is possible that a strong isohydric strategy in this species allowed survival despite an apparently unfavourable TLP value.

Interestingly, the LDMC also turned out to be a good predictor of species‐specific risk of decline. This might derive from correlations between leaf mechanical properties and drought tolerance, as found in some studies (Méndez‐Alonzo *et al*. 2019). In particular, low values of TLP have been associated with increased thickness and mechanical strength of cell walls, which in turn would affect LDMC. Similarly, leaf resistance to hydraulic failure under drought has been correlated to increased investment in biomass per unit leaf volume (Nardini *et al*. 2012), a pattern likely related to the role of leaf shrinkage in leaf hydraulic impairment (Scoffoni *et al*. 2014), whereby ‘hard’ leaves would suffer less shrinkage under water stress conditions. Nevertheless, the correlation found in this study is potentially useful, as LDMC is relatively easy to measure and might emerge as a good parameter for a first level screening of urban tree resilience.

The species‐specific risk of decline over multi‐annual surveys was also exponentially related to *P*_50_, another important proxy of plant tolerance to drought stress. This finding is consistent with several reports indicating drought‐induced xylem embolism and hydraulic failure as a major determinant of tree mortality (Anderegg *et al*. 2016). *P*_50_ is a parameter that is more difficult to measure than TLP, and techniques used to estimate the vulnerability to xylem embolism of different species can be prone to artefacts (Cochard *et al*. 2013; Wheeler *et al*. 2013; Trifilò *et al*. 2014; Nardini *et al*. 2017; Savi *et al*. 2017b). Nonetheless, reliable data on vulnerability to xylem embolism are now available for a large number of species from different biomes, including tree species frequently used for urban greening (Choat *et al*. 2012). Indeed, in the present study we did not measure *P*_50_ on tree individuals growing in Trieste but derived average values of this parameter from the literature (Table S2). Despite this approximation, the relationship between tree health status and *P*_50_ was highly indicative of the species‐specific risk of decline. This is a likely outcome of the limited intraspecific genotypic and phenotypic plasticity of vulnerability to xylem embolism (Wortemann *et al*. 2011; Lamy *et al*. 2014), which also represents a possible limitation to the capacity of urban trees to acclimate their hydraulic systems to ongoing climate changes (Savi *et al*. 2015). Moreover, our findings also suggest that values of *P*_50_ available in the literature might be used as important functional proxies that can be adopted in the selection of drought‐tolerant urban trees. In particular, under the climate conditions experienced by the town of Trieste over the last 8 years, only species with *P*_50_ < −3 MPa (breakpoint of the relationship between the Risk index and *P*_50_ equal to −3.2 ± 0.36 MPa) maintained a good health status, while more vulnerable species incurred a significant risk of dieback and decline, making them unsuitable candidates to substitute for dead trees or to implement in new plantations. Examples of such species to be preferred for urban sites in drought‐prone areas are *Q. ilex* and *Prunus mahaleb*, while species like *Robinia pseudoacacia* and *P. nigra* should be avoided.

Several studies have reported close correlations between *P*_50_ and WD (Pratt *et al*. 2007; Markesteijn *et al*. 2011; Nardini *et al*. 2013), and hence we expected to find a similar relationship in our dataset, as well as a significant relationship between species‐specific risk of decline and WD. However, WD was found to be independent on *P*_50_ values, and not related to the risk of decline of different tree species. While somewhat unexpected, similar outcomes have been reported previously (Savi *et al*. 2017a; Trueba *et al*. 2017), suggesting that the correlation between these two traits is not very strong nor general, but might depend on the features of the species’ assemblage considered. In our specific case, it has to be considered that urban trees comprise several different species, sometimes with very different geographic origins, that have been planted for their ornamental value. Our dataset, for example, included both angiosperm and gymnosperm species, mostly originating from temperate habitats (*e.g*. *T. cordata*) but mixed with taxa originating from dry sclerophyllous forests (*e.g*. *Q. ilex*) and even to alien species (*e.g*. *Ailanthus altissima*). Hence, urban trees represent a very heterogeneous assemblage from a biogeographic and phylogenetic point of view, likely subtending important structural differences at the wood level which might explain the lack of correlation between WD and *P*_50_. This is consistent with findings of Gleason *et al*. (2016), who found only a very weak correlation between WD and *P*_50_ across a global dataset comprising 335 angiosperm and 89 gymnosperm species. We call for more studies aimed at verifying the potential use of WD as a proxy for drought tolerance of urban trees. In fact, WD is easy to measure and can be highly replicated, making this trait an attractive potential proxy for selection of hardy urban trees.

In conclusion, our data reveal that urban trees prone to drought‐induced decline generally belong to species characterized by relatively high (less negative) values of TLP and *P*_50_, consistent with similar findings in natural forests worldwide (Anderegg *et al*. 2016). Notably, the correlation between these physiological traits and tree decline emerged regardless of untested effects of species‐specific water use strategy (iso‐ *versus* anisohydric, but see Klein 2014), phylogeny, heterogeneity of site‐specific environmental conditions and past management of crowns. Hence, while confirming the adaptive value of these physiological traits for woody plants growing in stressful environments, our findings offer a new perspective for management of urban trees and forests. In particular, species with low symplastic and apolastic tolerance to water shortage should be strictly monitored for future risks of decline, and new species to be planted should preferably be selected based on such sound physiological criteria, among others related to aesthetic and functional features.

## AUTHOR CONTRIBUTIONS

AN and GB designed the study and planned experiments. FPe, ET and ADB collected samples, performed experimental measurements and analysed the data. MT and SN contributed to experimental measurements. FPa provided the database of urban tree health status and contributed to the selection of experimental trees. AN and FPe wrote the manuscript, with contribution from all co‐authors.

## Supporting information


**Table S1**. Mean values and associated standard deviation of leaf dry matter content (LDMC), water potential at turgor loss point (TLP), water potential inducing 50% loss of hydraulic conductivity (*P*_50_) and wood density (WD) measured on the selected species. Note that for some species it was not possible to calculate the standard deviation of Ψ_50_ because only one value was available.
**Table S2**. List of the species selected in this study along with sources of *P*_50_ values.
**Table S3**. Summary of linear models run to assess the relationships between Risk index and LDMC, TLP, and WD and of the nonlinear least square (nls) model run to assess the relationship between Risk index and P_50_, measured for each species. SE =standard error.
**Table S4**. Summary of segmented analysis run to assess the breakpoint of the relationship between Risk index and P_50_ measured for each species. SE = standard error.
