# Supplementary material for: Turgor loss point and vulnerability to xylem embolism predict species‐specific risk of drought‐induced decline of urban trees
Source: Plant Biol (Stuttg). 2021 Oct 27;24(7):1198–207. doi: 10.1111/plb.13355 (PMC10078640; doi:10.1111/plb.13355)
Supplement: Supplementary file 1 — Table S1. Mean values and associated standard deviation of leaf dry matter content (LDMC), water potential at turgor loss point (TLP), water potential inducing 50% loss of hydraulic conductivity (P 50) and wood density (WD) measured on the selected species. Note that for some species it was not possible to calculate the standard deviation of Ψ50 because only one value was available. Table S2. List of the species selected in this study along with sources of P 50 values. Table S3. Summary of linear models run to assess the relationships between Risk index and LDMC, TLP, and WD and of the nonlinear least square (nls) model run to assess the relationship between Risk index and P50, measured for each species. SE =standard error. Table S4. Summary of segmented analysis run to assess the breakpoint of the relationship between Risk index and P50 measured for each species. SE = standard error. [file PLB-24-1198-s001.docx]

SUPPLEMENTARY MATERIALS

|  | Species | LDMC (mg g^-1^) | TLP (MPa) | P_50_ (MPa) | WD (g cm^-3^) |
| --- | --- | --- | --- | --- | --- |
| *Angiosperms* | *Styphnolobium japonicum* | 298.82±30.62 | -1.67±0.12 | -1.79±1.14 | 0.58±0.10 |
|  | *Populus nigra* | 346.60±29.34 | -2.14±0.22 | -1.45 | 0.46±0.05 |
|  | *Robinia pseudoacacia* | 343.75±28.21 | -1.89±0.18 | -2.15±0.74 | 0.64±0.14 |
|  | *Aesculus hippocastanum* | 296.62±42.61 | -1.39±0.24 | / | 0.38±0.04 |
|  | *Quercus pubescens* | 438.98±12.63 | -2.47±0.07 | -2.41±0.79 | 0.62±0.08 |
|  | *Tilia cordata* | 341.37±58.83 | -1.98±0.38 | -1.92±0.49 | 0.32±0.02 |
|  | *Juglans regia* | 307.51±32.37 | -1.68±0.14 | -2.14±0.34 | 0.38±0.02 |
|  | *Prunus mahaleb* | 325.24±35.13 | -2.27±0.15 | -5.18±0.25 | 0.74±0.07 |
|  | *Celtis australis* | 390.99±70.21 | -2.01±0.23 | / | 0.58±0.06 |
|  | *Cercis siliquastrum* | 298.75±24.41 | -1.65±0.17 | -1.80 | 0.45±0.06 |
|  | *Prunus cerasifera* | 326.76±21.98 | -2.21±0.18 | / | 0.70±0.10 |
|  | *Quercus ilex* | 533.80±39.79 | -3.09±0.38 | -4.03±1.37 | 0.70±0.08 |
|  | *Tamarix gallica* | 356.28±33.33 | -2.55±0.36 | / | 0.74±0.08 |
|  | *Ulmus minor* | 368.89±21.46 | -2.15±0.13 | -2.07±1.47 | 0.65±0.05 |
|  | *Platanus x acerifolia* | 291.47±39.33 | -1.58±0.16 | -1.75 | 0.52±0.11 |
|  | *Acer campestre* | 421.34±40.00 | -2.27±0.32 | -4.18±0.01 | 0.54±0.04 |
|  | *Fraxinus ornus* | 409.73±28.49 | -2.85±0.36 | -2.99±0.69 | 0.59±0.06 |
|  | *Ailanthus altissima* | 393.60±29.48 | -1.92±0.19 | -1.95±1.43 | 0.50±0.07 |
|  | *Ostrya carpinifolia* | 441.82±25.43 | -2.30±0.23 | -3.76±0.77 | 0.65±0.14 |
|  | *Tilia platyphyllos* | 341.37±46.51 | -2.18±0.30 | -3.39 | 0.34±0.05 |
|  | *Laurus nobilis* | 298.27±23.89 | -2.82±0.24 | -4.19±2.75 | 0.46±0.07 |
|  | *Ligustrum lucidum* | 437.18±54.25 | -2.13±0.50 | -3.67 | 0.57±0.07 |
|  | *Carpinus betulus* | 426.58±24.58 | -2.78±0.27 | -3.67 | 0.56±0.02 |
| *Gymnosperms* | *Ginkgo biloba* | 278.82±26.09 | -1.95±0.06 | / | 0.51±0.03 |
|  | *Pinus pinea* | 376.08±28.71 | -2.56±0.22 | -2.41±0.12 | 0.54±0.04 |
|  | *Cedrus deodara* | 387.34±20.16 | -2.57±0.42 | -4.27±0.96 | 0.83±0.22 |
|  | *Cedrus atlantica* | 415.97±46.12 | -2.82±0.47 | -4.55±0.08 | 0.78±0.17 |
|  | *Cupressus arizonica* | 385.55±27.46 | -2.19±0.15 | / | 0.70±0.08 |
|  | *Cupressus sempervirens* | 456.86±36.72 | -2.56±0.22 | / | 0.75±0.09 |
|  | *Pinus nigra* | 415.43±28.12 | -2.26±0.31 | -3.34±0.21 | 0.43±0.03 |
|  | *Pinus sylvestris* | 399.80±24.94 | -2.36±0.22 | -2.74±0.71 | 0.44±0.03 |
|  | *Cedrus libani* | 387.87±34.63 | -2.55±0.29 | / | 0.81±0.32 |

Tab S1. Mean values and associated standard deviation of leaf dry matter content (LDMC), water potential at turgor loss point (TLP), water potential inducing 50% loss of hydraulic conductivity (P_50_) and wood density (WD) measured on the selected species. Note that for some species it was not possible to calculate the standard deviation of Ψ_50_ because only one value was available.

Tab S2. List of the species selected in this study along with sources of P_50_ values.

|  | Species | References |
| --- | --- | --- |
| *Angiosperms* | *Styphnolobium japonicum* | Choat *et al.,* 2004; Dai *et al.,* 2020 |
|  | *Populus nigra* | De Baerdemaeker *et al.,* 2017 |
|  | *Robinia pseudoacacia* | Wang *et al.,* 2014; Chen *et al.,* 2019; Kiorapostolu *et al.,* 2019; Savi *et al.,* 2019; Dai *et al.,* 2020 |
|  | *Aesculus hippocastanum* | / |
|  | *Quercus pubescens* | Cochard *et al.,* 1992; Tognetti *et al.,* 1998; Tognetti *et al.,* 1999; Pita *et al.,* 2005; Domec *et al.,* 2017; Kiorapostolu *et al.,* 2019; Savi *et al.,* 2019 |
|  | *Tilia cordata* | Li *et al.,* 2008; Kiorapostolu *et al.,* 2019; Savi *et al.,* 2019 |
|  | *Juglans regia* | Tyree *et al.,* 1993; Chen *et al.,* 2019 |
|  | *Prunus mahaleb* | Savi *et al.,* 2017; Savi *et al.,* 2019 |
|  | *Celtis australis* | / |
|  | *Cercis siliquastrum* | Nardini *et al.,* 2003 |
|  | *Prunus cerasifera* | / |
|  | *Quercus ilex* | Lo Gullo & Salleo 1992; Tognetti *et al.,* 1998; Tognetti *et al.,* 1999; Martinez-Vilalta *et al.,* 2002; Pita *et al.,* 2005; Quero *et al.,* 2011; Limousin *et al.,* 2012; Pinto *et al.,* 2012; Peguero-Pina *et al.,* 2014; Trifilò *et al.,* 2015; Rodriguez-Calcerrada *et al.,* 2017; Domec *et al.,* 2017; Peguero-Pina *et al.,* 2018; Kiorapostolu *et al.,* 2019; Rosas *et al.,* 2019; Savi *et al.,* 2019 |
|  | *Tamarix gallica* | / |
|  | *Ulmus minor* | Venturas *et al.,* 2014 |
|  | *Platanus x acerifolia* | Kaack *et al.*, 2021 |
|  | *Acer campestre* | Rosner *et al.*, 2019; Savi *et al.*, 2019 |
|  | *Fraxinus ornus* | Tognetti *et al.,* 1999, Petruzzellis *et al.,* 2019; Savi *et al.,* 2019 |
|  | *Ailanthus altissima* | Chen *et al.,* 2019; Krӧber *et al.,* 2014; Petruzzellis *et al.,* 2019 |
|  | *Ostrya carpinifolia* | Kiorapostolu *et al.,* 2019; Savi *et al.,* 2019 |
|  | *Tilia platyphyllos* | Kaack *et al.*, 2021 |
|  | *Laurus nobilis* | Salleo *et al.,* 1996; Hacke & Sperry 2003; Klepsch *et al.,* 2016; Nardini *et al.,* 2017; Lamarque *et al.,* 2018 |
|  | *Ligustrum lucidum* | Fan *et al.,* 2018 |
|  | *Carpinus betulus* | Savi *et al.,* 2019 |
| *Gymnosperms* | *Ginkgo biloba* | / |
|  | *Pinus pinea* | Oliveras *et al.,* 2003; DM Johnson *et al.,* 2016 |
|  | *Cedrus deodara* | Cochard 1992; Brodribb *et al.*, 2014 |
|  | *Cedrus atlantica* | Cochard 1992; Pita *et al.,* 2005 |
|  | *Cupressus arizonica* | / |
|  | *Cupressus sempervirens* | / |
|  | *Pinus nigra* | Martinez-Vilalta & Piñol 2002; Rosas *et al.,* 2019 |
|  | *Pinus sylvestris* | Mencuccini *et al.,* 1997; Martinez-Vilalta & Piñol 2002;  Poyatos *et al.,* 2007; Aguadé *et al.,* 2015; Torres-Ruiz *et al.,* 2016; Jin *et al.,* 2018 |
|  | *Cedrus libani* | / |

| Risk Index | Estimate | SE | t-value | p-value |
| --- | --- | --- | --- | --- |
| Intercept | 1.23 | 0.40 | 3.10 | 0.004 |
| LDMC | -0.002 | 0.001 | -2.07 | 0.04 |
| R^2^ | 0.12 |  |  |  |
| Intercept | 1.26 | 0.33 | 3.78 | < 0.001 |
| TLP | 0.37 | 0.15 | 2.56 | 0.01 |
| R^2^ | 0.18 |  |  |  |
| Intercept | 0.50 | 0.28 | 1.77 | 0.09 |
| WD | -0.14 | 0.47 | -0.29 | 0.77 |
| R^2^ | 0.01 |  |  |  |
| P50 nls regression |  |  |  |  |
| a | 28.39 | 41.99 | 0.68 | 0.50 |
| b | 1.84 | 0.92 | 2.01 | 0.04 |
| c | 0.50 | 0.20 | 2.47 | 0.02 |
| pseudo R^2^ | 0.60 |  |  |  |

Tab. S3. Summary of linear models run to assess the relationships between Risk index and LDMC, TLP, and WD and of the nonlinear least square (nls) model run to assess the relationship between Risk index and P_50_, measured for each species. SE = standard error.

| Risk index | Estimate | SE | t-value | p-value |
| --- | --- | --- | --- | --- |
| Intercept | 1.23 | 0.40 | 3.10 | 0.004 |
| LDMC | -0.002 | 0.001 | -2.07 | 0.04 |
| R^2^ | 0.12 |  |  |  |

Tab. S4. Summary of segmented analysis run to assess the breakpoint of the relationship between Risk index and P_50_ measured for each species. SE = standard error.

P_50_ references:

**Aguadé D, Poyatos R, Gòmez M, Oliva J, Martínez-Vilalta J. 2015**. The role of defoliation and root rot pathogen infection in driving the mode of drought-related physiological decline in Scots pine (*Pinus sylvestris* L.). *Tree Physiology* **35**: 229-242.

**Brodribb TJ, McAdam SAM, Jordan GJ, Martin SCV. 2014**. Divergent adaptation to drought in conifers. *Proceedings of the National Academy of Sciences* **111**: 14489-14493.

**Chen Z, Li S, Luan J, Zhang Y, Zhu S, Wan X, Liu S. 2019**. Prediction of temperate broadleaf tree species mortality in arid limestone habitats with stomatal safety margins. *Tree Physiology* **39**: 1428-1437.

**Choat B, Jansen S, Zwieniecki MA, Smets E, Holbrook NM. 2004.** Changes in pit membrane porosity due to deflection and stretching: the role of vestured pits. *Journal of Experimental Botany* **55**: 1569-1575.

**Cochard H. 1992**. Vulnerability of several conifers to air embolism. *Tree Physiology* **11**: 73-83.

**Cochard H, Breda N, Granier A, Aussenac G. 1992**. Vulnerability to air-embolism of 3 European oak species (*Quercus petraea* (Matt) Liebl, *Quercus pubescens* Willd, *Quercus robur* L). *Annales des Sciences Forestières* **49**: 225-233.

**Dai Y, Wang L, Wan X. 2020**. Frost fatigue and its spring recovery of xylem conduits in ring-porous, diffuse-porous, and coniferous species in situ. *Plant Physiology and Biochemistry* **146**: 177-186.

**De Baerdemaeker NJF, Salomon RL, De Roo L, Steppe K. 2017.** Sugars from woody tissue photosynthesis reduce xylem vulnerability to cavitation. *New Phytologist* **216**: 720-727.

**Domec J-C, Smith DD, McCulloh KA. 2017.** A synthesis of the effects of atmospheric carbon dioxide enrichment on plant hydraulics: implications for whole‐plant water use efficiency and resistance to drought. *Plant, Cell & Environment* **40**: 921– 937.

**Fan D, Zhang S, Yan H, Wu Q, Xu X, Wang X. 2018.** Do karst woody plants control xylem tension to avoid substantial xylem cavitation in the wet season? *Forest Ecosystems* **5**, 40.

**Jin Y, Wang C, Zhou Z. 2018.** Conifers but not angiosperms exhibit vulnerability segmentation between leaves and branches in a temperate forest. *Tree Physiology* **39**: 454-462.

**Johnson DM, Wortemann R, McCulloh KA, Jordan-Meille L, Ward E, Warren JM, Palmroth S, Domec J-C 2016.** A test of the hydraulic vulnerability segmentation hypothesis in angiosperm and conifer tree species. *Tree Physiology* **36**: 983-993.

**Kaack L, Weber M, Isasa E, Karimi Z, Li S, Pereira L, Trabi CL, Zhang Y, Schenk HJ, Schuldt B, Schmidt V, Jansen S 2021.** Pore constrictions in intervessel pit membranes provide a mechanistic explanation for xylem embolism resistance in angiosperms. *New Phytologist* **230**: 1829-1843.

**Kiorapostolou N, Da Sois L, Petruzzellis F, Savi T, Trifilò P, Nardini A, Petit G. 2019.** Vulnerability to xylem embolism correlates to wood parenchyma fraction in angiosperms but not in gymnosperms. *Tree Physiology* **39**: 1675-1684.

**Klepsch M, Zhang Y, Kotowska M, Lamarque LJ, Nolf M, Schuldt B, Torres-Ruiz JM, Qin D-W, Choat B, Delzon S, Scoffoni C, Cao K-F, Jansen S. 2016.** Is xylem of angiosperm leaves less resistant to embolism than branches? Insights from microCT, hydraulics, and anatomy. *Journal of Experimental Botany* **22**: 5611-5623.

**Kröber W, Zhang S, Ehmig M, Bruelheide H. 2014.** Linking Xylem Hydraulic Conductivity and Vulnerability to the Leaf Economics Spectrum—A Cross-Species Study of 39 Evergreen and Deciduous Broadleaved Subtropical Tree Species. *PLoS ONE* **9**: e109211.

**Hacke UG, Sperry JS. 2003.** Limits to xylem refilling under negative pressure in *Laurus nobilis* and *Acer negundo*. *Plant, Cell & Environment* **26**: 303-311.

**Lamarque LJ, Corso D, Torres-Ruiz JM, Badel E, Brodribb TJ, Burlett R, Charrier G, Choat B, Cochard H, Gambetta GA et al. 2018.** An inconvenient truth about xylem resistance to embolism in the model species for refilling *Laurus nobilis* L. *Annals of Forest Science* **75**, 88.

**Martínez-Vilalta J, Piñol J. 2002.** Drought-induced mortality and hydraulic architecture in pine populations of the NE Iberian Peninsula. *Forest Ecology and Management* **161**: 247-256.

**Li Y, Sperry JS, Taneda H, Bush SE, Hacke UG. 2008**. Evaluation of centrifugal methods for measuring xylem cavitation in conifers, diffuse- and ring-porous angiosperms. *New Phytologist* **177**: 558-568.

**Limousin JM, Longepierre D, Huc R, Rambal S. 2010.** Change in hydraulic traits of Mediterranean *Quercus ilex* subjected to long-term throughfall exclusion. *Tree Physiology* **30**: 1026–1036.

**Lo Gullo MA, Salleo S. 1992.** Water storage in the wood and xylem cavitation in 1-year-old twigs of *Populus deltoides* Bartr. *Plant, Cell & Environment* **15**: 431-438.

**Martínez-Vilalta J, Prat E, Oliveras I, Piñol J. 2002.** Xylem hydraulic properties of roots and stems of nine Mediterranean woody species. *Oecologia* **133**: 19-29.

**Mencuccini M, Grace J, Fioravanti M. 1997.** Biomechanical and hydraulic determinants of tree structure in Scots pine: anatomical characteristics. *Tree Physiology* **17**: 105-113.

**Nardini A, Salleo S, Raimondo F. 2003.** Changes in leaf hydraulic conductance correlate with leaf vein embolism in *Cercis siliquastrum* L. *Trees* **17**: 529-534.

**Nardini A, Savi T, Losso A, Petit G, Pacilè S, Tromba G, Mayr S, Trifilò P, Lo Gullo M.A., Salleo S. 2017.** X‐ray microtomography observations of xylem embolism in stems of *Laurus nobilis* are consistent with hydraulic measurements of percentage loss of conductance. *New Phytologist* **213**: 1068-1075.

**Oliveras I, Martínez-Vilalta J, Jimenez-Ortiz T, Lledó MJ, Escarré A, Piñol J. 2003**. Hydraulic properties of *Pinus halepensis*, *Pinus pinea* and *Tetraclinis articulata* in a dune ecosystem of Eastern Spain. *Plant Ecology* **169**: 131-141.

**Peguero-Pina JJ, Sancho-Knapik D, Barrón E, Camarero JJ, Vilagrosa A, Gil-Pelegrín E. 2014.** Morphological and physiological divergences within *Quercus ilex* support the existence of different ecotypes depending on climatic dryness. *Annals of Botany* **114**: 301–313.

**Peguero-Pina JJ, Mendoza-Herrer Ó, Gil-Pelegrín E, Sancho-Knapik D. 2018.** Cavitation Limits the Recovery of Gas Exchange after Severe Drought Stress in Holm Oak (*Quercus ilex* L.). *Forests* **9**:443.

**Petruzzellis F, Nardini A, Savi T, Tonet V, Castello M, Bacaro G. 2019.** Less safety for more efficiency: water relations and hydraulics of the invasive tree *Ailanthus altissima* (Mill.) Swingle compared with native *Fraxinus ornus* L. *Tree Physiology* **39**: 76-87.

**Pinto CA, David JS, Cochard H, Caldeira MC, Henriques MO, Quilhó T, Paço TA, Pereira JS, David T.S. 2012.** Drought-induced embolism in current-year shoots of two Mediterranean evergreen oaks. *Forest Ecology and Management* **285**: 1-10.

**Pita P, Cañas I, Soria F, Ruiz F, Toval G. 2005.** Use of physiological traits in tree breeding for improved yield in drought-prone environments. The case of *Eucalyptus globulus*. *Investigación agraria. Sistemas y recursos forestales* **14**: 383-393.

**Poyatos R, Martínez-Vilalta J, Čermák J, Ceulemans R, Granier A, Irvine J, Kӧstner B, Lagergren F, Meiresonne L, Nadezhdina N et al. 2007.** Plasticity in hydraulic architecture of Scots pine across Eurasia. *Oecologia* **153**, 245–259.

**Quero JL, Sterck F, Martinez-Vilalta J, Villar R. 2011.** Water-use strategies of six co-existing Mediterranean woody species during a summer drought. *Oecologia* **166**: 45-57.

**Rodríguez‐Calcerrada J, Li M, López R, Cano FJ, Oleksyn J, Atkin OK, Pita P, Aranda I, Gil L. 2017.** Drought‐induced shoot dieback starts with massive root xylem embolism and variable depletion of nonstructural carbohydrates in seedlings of two tree species. *New Phytologist* **213**: 597-610.

**Rosas T, Mencuccini M, Barba J, Cochard H, Saura‐Mas S, Martínez‐Vilalta J. 2019.** Adjustments and coordination of hydraulic, leaf and stem traits along a water availability gradient. *New Phytologist* **223**: 632-646.

**Rosner S, Heinze B, Savi T, Dalla‐Salda G. 2019.** Prediction of hydraulic conductivity loss from relative water loss: new insights into water storage of tree stems and branches. *Physiologia Plantarum* **165**: 843-854.

**Salleo S, Gullo MAL, De Paoli D, Zippo M. 1996.** Xylem recovery from cavitation‐induced embolism in young plants of *Laurus nobilis*: a possible mechanism. *New Phytologist* **132**: 47-56.

**Savi T, Love VL, Dal Borgo A, Martellos S, Nardini A. 2017.** Morpho-anatomical and physiological traits in saplings of drought-tolerant Mediterranean woody species. *Trees* **31**: 1137-1148.

**Savi T, Tintner J, Da Sois L, Grabner M, Petit G, Rosner S. 2019.** The potential of Mid-Infrared spectroscopy for prediction of wood density and vulnerability to embolism in woody angiosperms. *Tree Physiology* **39**: 503–510.

**Tognetti R, Longobucco A, Raschi A. 1998.** Vulnerability of xylem to embolism in relation to plant hydraulic resistance in *Quercus pubescens* and *Quercus ilex* co-occurring in a Mediterranean coppice stand in central Italy. *New Phytologist* **139**: 347-448.

T**ognetti R, Longobucco A, Raschi A. 1999.** Seasonal embolisms and xylem vulnerability in deciduous and evergreen Mediterranean trees influenced by proximity to a carbon dioxide spring. *Tree Physiology* **19**: 271-277.

**Torres‐Ruiz JM, Cochard H, Mencuccini M, Delzon S, Badel E.** 2016. Direct observation and modelling of embolism spread between xylem conduits: a case study in Scots pine. *Plant, Cell & Environment* **39**: 2774– 2785.

**Trifilò P, Nardini A, Lo Gullo MA, Barbera PM, Savi T, Raimondo F. 2015.** Diurnal changes in embolism rate in nine dry forest trees: relationships with species-specific xylem vulnerability, hydraulic strategy and wood traits. *Tree Physiology* **7**: 694-705.

**Tyree MT, Cochard H, Cruiziat P, Sinclair B, Ameglio T. 1993.** Drought-induced leaf shedding in walnut: evidence for vulnerability segmentation. *Plant, Cell & Environment* **16**: 879-882.

**Venturas M, López R, Martín JA, Gascó A, Gil L. 2014**. Heritability of *Ulmus minor* resistance to Dutch elm disease and its relationship to vessel size, but not to xylem vulnerability to drought. *Plant Pathology* **63**: 500-509.

**Wang R, Zhang L, Zhang, S, Cai J, Tyree MT. 2014.** Water relations of *Robinia pseudoacacia* L.: do vessels cavitate and refill diurnally or are R‐shaped curves invalid in *Robinia*? *Plant Cell & Environment* **37**: 2667-2678.
